# Online Monitoring and Fault Diagnosis for High-Dimensional Stream with Application in Electron Probe X-Ray Microanalysis

**DOI:** 10.3390/e27030297

**Published:** 2025-03-13

**Authors:** Tao Wang, Yunfei Guo, Fubo Zhu, Zhonghua Li

**Affiliations:** 1 School of Mathematics and Statistics, Huaiyin Normal University, Huai’an 223300, China; 8201711075@hytc.edu.cn (T.W.); 8200211060@hytc.edu.cn (F.Z.); 2 Department of Mathematics, Yanbian University, Yanji 133002, China; guoyunfei0413@ybu.edu.cn; 3 School of Statistics and Data Science, LPMC, LEBPS and KLMDASR, Nankai University, Tianjin 300071, China

**Keywords:** change point detection, extreme value theory, fault diagnosis, high-dimensional statistics, max-norm information, X-ray microanalysis

## Abstract

This study introduces an innovative two-stage framework for monitoring and diagnosing high-dimensional data streams with sparse changes. The first stage utilizes an exponentially weighted moving average (EWMA) statistic for online monitoring, identifying change points through extreme value theory and multiple hypothesis testing. The second stage involves a fault diagnosis mechanism that accurately pinpoints abnormal components upon detecting anomalies. Through extensive numerical simulations and electron probe X-ray microanalysis applications, the method demonstrates exceptional performance. It rapidly detects anomalies, often within one or two sampling intervals post-change, achieves near 100% detection power, and maintains type-I error rates around the nominal 5%. The fault diagnosis mechanism shows a 99.1% accuracy in identifying components in 200-dimensional anomaly streams, surpassing principal component analysis (PCA)-based methods by 28.0% in precision and controlling the false discovery rate within 3%. Case analyses confirm the method’s effectiveness in monitoring and identifying abnormal data, aligning with previous studies. These findings represent significant progress in managing high-dimensional sparse-change data streams over existing methods.

## 1. Introduction

In recent years, the rapid advancement of modern sensing and data acquisition technologies has enabled the real-time collection of high-dimensional data streams across diverse industrial applications. Such data streams often exhibit shifts in statistical properties, transitioning from an in-control (IC) state to an out-of-control (OC) state due to anomalies. For instance, in electron probe X-ray microanalysis, real-time monitoring of compositional changes in materials requires the detection of subtle shifts in high-dimensional spectral data, which may occur either abruptly or gradually. Addressing such scenarios requires robust online monitoring and fault diagnosis methods tailored for high-dimensional and sparsely changing data streams.

**Figure 1:**
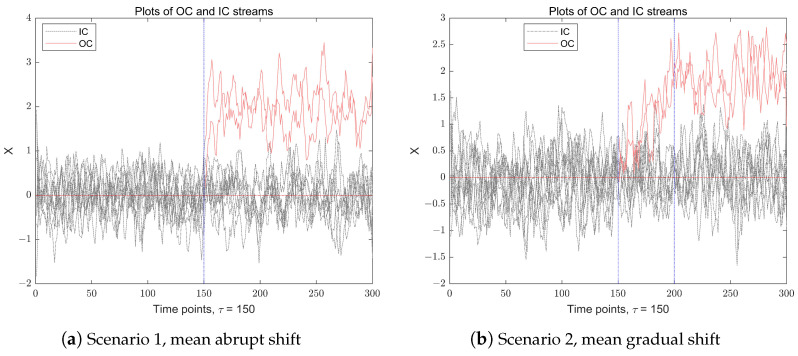
The data stream with IC data (gray) and OC data (red), when p=10, $p_{s}=2$, τ=150, t=300, κ=2.0. The left subfigure is the mean abrupt shift case, and the right subfigure is the mean gradual shift case.

Before proceeding, for an intuitive explanation, we present a simple example to illustrate IC and OC observations in a multidimensional data stream. Figure 1 illustrates a *p*-dimensional (p=10) time series data stream recorded over t=300 time points. The IC observations, denoted as $X_{i}$, are independently and identically distributed (i.i.d.) multivariate normal random variables with the mean vector $\mu_{0}={\left(0,0,\ldots ,0\right)}_{p}^{\prime }$, and a covariance matrix $\Sigma_{p}=I_{p}$. After a change point τ=150, the $p_{s}=2$ components of the observations experience an anomaly, resulting in a shift in the data stream. The figure presents two distinct scenarios of mean-shift anomalies in a multidimensional data stream: the left subfigure depicts a mean abrupt shift scenario, where the change occurs suddenly after a time point τ, while the right subfigure illustrates a mean gradual shift scenario, where the shift changes in a duration of about a 50-time-point interval, ultimately stabilizing when t>200.

In the examples mentioned, the change points in the data stream are easily identifiable due to the low dimensionality, allowing easy visual detection. However, in real-world applications, monitoring complex and high-dimensional data streams is more challenging. Anomalies may only appear in a small subset of variables at any given time, and the intricate interactions and high dimensionality of these variables complicate online monitoring and fault detection. This is especially true when pinpointing the exact time and specific elements that show abnormal variations. For instance, in semiconductor manufacturing, subtle sensor anomalies may indicate critical equipment issues, while in energy infrastructure, gradual pressure drifts could signal potential failures. Timely detection and diagnosis of such anomalies are crucial for operational safety and efficiency. Yet, the high dimensionality, sparsity of changes, and complex variable interdependencies present significant challenges to existing monitoring and diagnostic frameworks.

In order to overcome these challenges, a series of articles have been developed on the monitoring and diagnosis of high-dimensional data streams. Here, we roughly divide them into the following three categories.

(1) Statistical and Control Chart-Based Methods. Statistical and control chart-based methods are widely employed for monitoring high-dimensional data streams, with prominent techniques including Hotelling’s $T^{2}$, Multivariate Exponentially Weighted Moving Average (MEWMA), Multivariate Cumulative Sum (MCUSUM), and principal component analysis (PCA)-based monitoring. Zou et al. (2015) [1] introduce a robust control chart utilizing local CUSUM statistics, demonstrating effective detection capabilities across both sparse and dense scenarios, albeit with certain limitations in parameter assumptions and implementation complexity. Ebrahimi et al. (2021) [2] developed an adaptive PCA-based monitoring method incorporating compressed sensing principles and adaptive lasso for change source identification, though its computational demands may be substantial for large-scale datasets. Li (2019) [3] proposes a flexible two-stage monitoring procedure with user-defined IC average run length (ARL) and type-I error rates, which requires meticulous calibration of control limits for both stages. For comprehensive reviews of online monitoring and diagnostic methods based on statistical process control (SPC), refer to studies such as [4,5,6,7,8,9,10,11,12].

(2) Information-Theoretic and Entropy-Based Methods. Entropy-based measures, including Shannon entropy, Renyi entropy, and approximate entropy, are widely used to assess the complexity and randomness of data streams in monitoring and diagnostics. Recent research has made significant progress in both theoretical and practical aspects of this field. Mutambik (2024) [13] develops E-Stream, an entropy-based clustering algorithm for real-time high-dimensional Internet of Things (IoT) data streams. By incorporating an entropy-based feature ranking method within a sliding window framework, the algorithm reduces dimensionality, improving clustering accuracy and computational efficiency. However, its dependence on manual parameter tuning restricts its adaptability to diverse datasets. Wan et al. (2024) [14] propose an entropy-based method for monitoring pressure pipelines using acoustic signals. Their approach combines Denoising Autoencoder (DAE) and Generative Adversarial Network (GAN) with entropy-based loss functions for noise reduction. While effective, the adversarial training process requires substantial computational resources, limiting its feasibility for real-time applications in resource-constrained environments. For a deeper understanding of recent advancements in entropy-based theories, methods, and applications for data stream monitoring and diagnostics, see [15,16,17,18,19]. These studies highlight the latest developments in this area.

(3) Machine Learning and Data-Driven Methods. In recent years, machine learning and data-driven methods have become increasingly important for monitoring and diagnostics in various fields. Online learning techniques, such as Online Support Vector Machines (OSVMs), Online Random Forests, and Stochastic Gradient Descent (SGD), have been successfully applied to real-time monitoring tasks. Additionally, deep learning models like Recurrent Neural Networks (RNNs), Long Short-Term Memory (LSTM) networks, and autoencoders have shown strong performance in capturing temporal patterns and detecting anomalies in high-dimensional data streams. Recent developments in this area include OLFA (Online Learning Framework for sensor Fault diagnosis Analysis) by Yan et al. (2023) [20], which provides a solution for real-time fault diagnosis in autonomous vehicles by addressing the non-stationary nature of sensor faults. Chen et al. (2024) [21] present a real-time fault diagnosis method for multisource heterogeneous information fusion based on two-level transfer learning (TTDNN), focusing on gearbox dataset, bearing dataset, etc. Another important contribution is from Li et al. (2025) [22], who propose a novel framework combining Deep Reinforcement Learning (DRL) with SPC for online monitoring of high-dimensional data streams under limited resources. Although their approach shows good scalability through deep neural networks, the computational demands of the Double Dueling Q-network training process may be a limitation for real-time applications with strict computational constraints. For comprehensive reviews of machine learning-based online monitoring and diagnostic methods, see studies such as [21,23,24,25,26,27].

Despite these advancements, three key limitations remain. First, many SPC methods face computational challenges in ultra-high dimensions due to reliance on covariance matrix inversions or extensive hypothesis testing. Second, entropy-based techniques, though effective for noise reduction, show limited sensitivity to sparse changes where only few variables deviate, while machine learning approaches often demand substantial labeled data that may be unavailable. Third, current diagnostic procedures typically emphasize either speed or accuracy, struggling to achieve both real-time alerts and precise root-cause identification. This paper bridges these gaps by proposing a two-stage monitoring and diagnostic framework designed for high-dimensional data streams with sparse mean shifts. The first issue is online monitoring, which aims to check whether the data stream has changed over time points and accurately estimate the change point τ after the change occurs. The second issue is the diagnosis of the fault, which aims to identify the components that have undergone abnormal changes; it will help eliminate the root causes of the change.

As the EWMA statistic is sensitive to small changes and includes the information of previous samples, we use it for online monitoring [28]. At the first stage, we construct a monitoring statistic based on the EWMA statistic. Under certain conditions, we prove the asymptotic distribution of the monitoring statistic and then obtain the threshold of online monitoring. At any time point, if the monitoring statistic value is greater than the threshold, a real-time alert is issued, indicating that the data stream is out of control. Then, we move into the second stage, to identify the components that have undergone abnormal changes over time points, which belongs to the scope of fault diagnosis. Specifically, when the monitoring procedure proposed in the first stage alerts that the data stream is abnormal, we allow the monitoring procedure to continue running for a while, and obtain more sample data, then we conduct multiple hypothesis tests on the data before and after the alarm to determine which components of the data stream have experienced anomalies. The desired two-stage monitoring and diagnosis scheme in this study can not only correctly estimate the time point in time but also accurately identify the OC components of the data stream.

The contributions of this paper are as follows: (1) Methodological Innovation: This study introduces an EWMA-based monitoring statistic that leverages max-norm aggregation to enhance sensitivity to sparse changes. After its asymptotic distribution is derived, a dynamic thresholding mechanism is established for real-time anomaly detection. (2) Integrated Diagnosis: Upon detecting an OC state, the framework employs a delayed multiple hypothesis testing procedure to isolate anomalous components. This approach minimizes false discoveries while accommodating temporal dependencies in post-alarm data. (3) Industrial Applicability: Our method is validated through electron probe X-ray microanalysis (EPXMA), a critical technique in materials science for real-time composition monitoring. The method’s efficiency in handling high-dimensional sparse shifts makes it equally viable for IoT-enabled predictive maintenance and industrial process control.

This paper is structured as follows: Section 2 formulates the monitoring and diagnosis problem, detailing the EWMA statistic, fault isolation strategy, and performance metrics. Section 3 evaluates the method via simulations, benchmarking against state-of-the-art techniques. Section 4 demonstrates its practicality through an EPXMA case study, highlighting its superiority in detecting micron-scale material defects. Conclusions and future directions are presented in Section 5.

## 2. Methodology

### 2.1. Problem Formulation

Consider a high-dimensional data stream denoted by $X_{1},X_{2},\ldots ,X_{t},\ldots$, where each observation $X_{t}={\left(x_{1t},x_{2t},\ldots ,x_{pt}\right)}^{\prime }\in R^{p}$ denotes a *p*-dimensional vector at time point *t*. In the IC state, the observations are generalized from $N(\mu_{0},\Sigma_{p})$, with $\mu_{0}={\left(\mu_{01},\ldots ,\mu_{0p}\right)}^{\prime }\in R^{p}$ and $\Sigma_{p}={\left(\sigma_{jk}\right)}_{p\times p}$, j,k=1,…,p. However, following an unknown change point τ, the process experiences a mean shift from $\mu_{0}$ to $\mu_{a}$, transitioning to an OC state. In practical applications, process changes are typically driven by only a small subset of components within the data stream. Consequently, the shift vector $\mu_{a}-\mu_{0}$ exhibits sparsity, with most elements being zero and only a few non-zero components. It is important to note that the transition from $\mu_{0}$ to $\mu_{a}$ can occur either abruptly or gradually, representing two distinct scenarios of mean-shift anomalies: abrupt mean shifts and gradual mean shifts.

This study aims to address two critical challenges in high-dimensional data stream monitoring: The first one is to propose an effective online monitoring method that can accurately estimate the location of the change point. Once the data stream changes abnormally, it can issue an alarm in time. The second task is to accurately identify the components of abnormal changes in high-dimensional data streams while controlling the error rate.

### 2.2. The EWMA Statistic

As is well known, the EWMA statistic is sensitive to small changes, and it includes information from previous samples [29]. Thus, an EWMA statistic is chosen in this study to monitor a high-dimensional data stream, the EWMA sequence is defined by

(1) $$Y_{t}=\gamma \left(X_{t}-\mu_{0}\right)+\left(1-\gamma \right)Y_{t-1},\;\;t=1,2,\ldots$$

where γ∈[0,1] is a weigh parameter. As described in Feng et al. (2020) [30], a smaller value of γ leads to a quicker detection of smaller shifts, and the detailed comparison results are provided in the numerical simulation section.

Let $Y_{t}={\left(y_{1t},y_{2t},\ldots ,y_{pt}\right)}^{\prime }$, the *j*-th component of $Y_{t}$ is denoted as

(2) $$y_{jt}=\gamma \left(x_{jt}-\mu_{0j}\right)+\left(1-\gamma \right)y_{j(t-1)},\;j=1,\ldots ,p,\;t=1,2,\ldots ,\;$$

where $Y_{0}={\left(0,0,\ldots ,0\right)}^{\prime }$ is prearranged, i.e., $y_{j0}=0$ for j=1,…,p. It is obvious that when the data stream is IC, we have

$$E\left(Y_{t}\right)={\left(0,0,\ldots ,0\right)}^{\prime },\;\;\;\;Cov\left(Y_{t}\right)=\left(\frac{\gamma }{2-\gamma }\right)\left(1-{\left(1-\gamma \right)}^{2t}\right)\Sigma .$$

As t→∞, we have $(1-{\left(1-\gamma \right)}^{2t})\to 1$, then the asymptotic distribution of $Y_{t}$ is as follows,

(3) $$Y_{t}∼N_{p}\left(0,\frac{\gamma }{2-\gamma }\Sigma \right),\;\;t\to \infty .$$

Correspondingly, the *j*-th component of $Y_{t}$ obeys the following normal distribution, i.e.,

$$y_{jt}∼N\left(0,\frac{\gamma }{2-\gamma }\sigma_{jj}\right),\;\;t\to \infty ,$$

where $\sigma_{jj}$ is the *j*-th diagonal element of Σ. However, once the mean value has undergone abnormal changes, $Y_{t}$ does not follow the multivariate normal distribution mentioned above.

### 2.3. Online Monitoring Procedure

Without loss of generality, we assume that the mean vectors of $Y_{1}$, $Y_{2}$, $Y_{3}$, …, $Y_{t}$, …mentioned above are $\mu_{1}$, $\mu_{2}$, …, $\mu_{t}$,…, respectively. Thus, the online monitoring of high-dimensional data streams can be considered as a hypothesis testing change point detection problem, with the null and the alternative hypothesis are

(4) $$\begin{array}{cc} \; & H_{0t}:\mu_{1}=\mu_{2}=,\ldots ,=\mu_{t}=0,\\ \; & H_{1t}:\exists \;\tau >0,\;\;\mu_{1}=\mu_{2}=,\ldots ,=\mu_{\tau -1}=0,\\ \; & \;\;\mathrm{but}\;\;\mu_{\tau +1},\ldots ,\mu_{t}\;\;\mathrm{are}\;\;\mathrm{not}\;\;\mathrm{equal}\;\;\mathrm{to}\;\;0. \end{array}$$

It is obvious that the monitored data stream is IC up to time point *t* if $H_{0t}$ is accepted, but a rejection of $H_{0t}$ indicates that some components of the stream have changed abnormally after the change point τ.

Therefore, we construct the test statistic as the following max-norm information:

(5) $$M_{t}=\max_{1\leq j\leq p}\frac{y_{jt}^{2}}{\frac{\gamma }{2-\gamma }\sigma_{jj}},\;\;t=1,2,\ldots .$$

Intuitively, the larger the value of $M_{t}$, the more likely the observation undergoes abnormal at time point *t*. If $M_{t}$ exceeds a threshold, it implies that an anomaly occurred.

It should be noted that the mean and covariance values of $Y_{t}$ are unknown, so the reasonable estimators of μ and Σ are needed. Suppose $X_{1},X_{2},\ldots ,X_{m}$ be the initial IC data stream, the sample mean and covariance of these *m* observations are defined as ${\hat{\mu }}_{0}=\frac{1}{m}\sum_{t=1}^{m}X_{t}$ and $\hat{\Sigma }=\frac{1}{m-1}\sum_{t=1}^{m}\left(X_{t}-{\hat{\mu }}_{0}\right){\left(X_{t}-{\hat{\mu }}_{0}\right)}^{\prime }$, respectively.

Consequently, the estimated max-normal monitoring statistic is defined as

(6) $${\tilde{M}}_{t}=\max_{1\leq j\leq p}\frac{y_{jt}^{2}}{\frac{\gamma }{2-\gamma }{\hat{\sigma }}_{jj}},\;\;t=m+1,m+2,\ldots .$$

where ${\hat{\sigma }}_{jj}$ is the *j*-th main diagonal element of $\hat{\Sigma }$.

For simplicity, let $z_{jt}=\frac{y_{jt}}{\sqrt{\frac{\gamma }{2-\gamma }{\hat{\sigma }}_{jj}}}$, j=1,…,p, it is obvious that, $z_{jt}∼N\left(0,1\right)$, t→∞. The monitoring statistic can be rewritten as

(7) $${\tilde{M}}_{t}=\max_{1\leq j\leq p}z_{jt}^{2}.$$

Let $Z_{t}={\left(z_{1t},z_{2t},\ldots ,z_{pt}\right)}^{\prime }$, then $Z_{t}$ is a high-dimensional normal random vector with the mean vector of zero and the covariance matrix of $\Omega ={\left(\omega_{jk}\right)}_{p\times p}$ for 1≤j,k≤p, where the diagonal $\omega_{jj}=1$ for 1≤j≤p. In order to obtain the threshold of online monitoring, we need to prove the asymptotic distribution of the statistic ${\tilde{M}}_{t}$ when the null hypothesis holds.

To this end, this requires the following two assumptions:

**A.1.**  *$\max_{1\leq j\neq k\leq p}\left|\omega_{i,j}\right|\leq r<1$ for some constant 0<r<1;*

**A.2.**  *$\max_{1\leq j\neq k\leq p}\omega_{i,j}^{2}\leq C_{0}$ for some constant $C_{0}$.*

Assumptions (A.1) and (A.2) are mild and common assumptions that are widely used in the high-dimensional means test under dependence. Under the above two assumptions, Proposition 1 studies the asymptotic distribution of ${\tilde{M}}_{t}$ when the null hypothesis holds.

**Proposition** **1.**  *Under the assumptions of (A.1) and (A.2), when the null hypothesis $H_{0t}$ holds, for any z∈R, ${\tilde{M}}_{t}$ follows the asymptotic distribution*

(8) $$P\left({\tilde{M}}_{t}-2\log\left(p\right)+\log\left(\log\left(p\right)\right)\leq z\right)\to \exp\left\{-\frac{1}{\sqrt{\pi }}\exp\left\{-\frac{z}{2}\right\}\right\},\;\;\;\mathrm{as}\;\;t,p\to \infty .$$

Proposition 1 shows that ${\tilde{M}}_{t}-2\log\left(p\right)+\log\left(\log\left(p\right)\right)$ follows the type I extreme value distribution with the cumulative distribution function $\exp\{-\frac{1}{\sqrt{\pi }}\exp\left\{-\frac{z}{2}\right\}\}$. The proof of this proposition is similar to that of Lemma 6 in Cai et al. (2014) [31], so it is omitted here.

Thus, the data stream shows abnormal change at time point *t*, if

(9) $${\tilde{M}}_{t}>L_{\alpha },\;$$

where $L_{\alpha }=2\log p-\log\left(\log p\right)+q_{\alpha }$ is the threshold at a given significance level α, and $q_{\alpha }=-\log\left(\pi \right)-2\log\left(\log{\left(1-\alpha \right)}^{-1}\right).$

It should be noted that not all the observations of ${\tilde{M}}_{t}>L_{\alpha }$ are considered as change points, and these points may also be outliers. Therefore, when ${\tilde{M}}_{t}>L_{\alpha }$ occurs, we continue to monitor for a while to obtain some more observations and if all the values of ${\tilde{M}}_{t}$ are greater than $L_{\alpha }$. Thus, the change point τ can be estimated naturally by

(10) $$\hat{\tau }=\inf\left\{t:t>t_{\mathrm{ic}}|{\tilde{M}}_{t+i}>L_{\alpha },\;i=0,1,\ldots ,n\right\}.$$

In this case, we claim that the data stream changes abnormally after time point τ, and the process enters into an OC state.

### 2.4. Fault Diagnosis Procedure

To this end, we first keep monitoring a *p*-dimensional data stream by using the monitoring statistics ${\tilde{M}}_{t}$. After the OC alarm is issued, we enter into the second stage of statistical fault diagnosis to identify the components that have undergone abnormal changes. To this end, we continue to monitor the data stream for a while and collect some more OC observations. The diagnostic problem can be regarded as the following multiple hypothesis testing. The null hypothesis and the alternative hypothesis are

(11) $$H_{0jt}:\mu_{1j}-\mu_{0j}=0,\;\;v.s.\;\;H_{1jt}:\mu_{1j}-\mu_{0j}\neq 0,\;\;\mathrm{for}\;\;j=1,\ldots ,p,\;\;t>\hat{\tau }.$$

It is obvious that if $H_{0jt}$ is accepted, the *j*-th component of the stream is IC; otherwise, a rejection $H_{0jt}$ indicates that the *j*-th component of the stream is OC when $t>\hat{\tau }$.

To address this problem, a diagnostic statistic ${\tilde{W}}_{jt}$ is proposed in the following form,

(12) $${\tilde{W}}_{jt}=\frac{{\bar{y}}_{jt}^{2}}{\frac{\gamma }{n(2-\gamma )}{\hat{\sigma }}_{jj}},\;\;j=1,\ldots ,p,\;\;t>\hat{\tau },$$

where ${\bar{y}}_{jt}=\frac{1}{n}\sum_{t=\hat{\tau }+1}^{\hat{\tau }+n}y_{it}$ is the mean value of the observed samples, and the sample variance ${\hat{\sigma }}_{jj}$ is defined as that in Equation (6). It can be seen that if the value of ${\tilde{W}}_{jt}$ is large, it indicates that abnormal changes may occur in the data stream. Therefore, it can be inferred that the null hypothesis in Equation (11) is rejected if ${\tilde{W}}_{jt}$ too large to exceed a threshold.

In many applied multiple testing problems, there are many test strategies, such as the traditional critical value test methods and some false discovery rate (FDR) controlling procedures [32,33,34]. It is obvious that $E({\bar{y}}_{jt})=0$, $Var\left({\bar{y}}_{jt}\right)=\frac{\gamma }{n(2-\gamma )}{\hat{\sigma }}_{jj}$, and $\frac{{\bar{y}}_{jt}}{\sqrt{\frac{\gamma }{n(2-\gamma )}{\hat{\sigma }}_{jj}}}∼N\left(0,1\right)$ as n→∞ under the null hypothesis $H_{0jt}$. Thus, the diagnostic statistic ${\tilde{W}}_{jt}$ follows Chi-squared distribution

(13) $${\tilde{W}}_{jt}\to \chi^{2}\left(1\right),\;\;\;\mathrm{as}\;\;n\to \infty .$$

In the diagnosis process, we should ensure that the global per-comparison error rate (PCER) can be controlled within a prespecified level α. Without loss of generality, we assume the first $p_{0}$ components of the high-dimensional observations are IC, and the remaining $p-p_{0}$ components are OC when t=τ+1,τ+2,…,τ+n. The PCER can be defined as the expected number of type-I errors divided by the number of hypotheses, that is,

(14) $${\mathrm{PCER}}_{t}=\frac{1}{p}\sum_{j=1}^{p_{0}}P_{H_{1t}}\left({\tilde{W}}_{jt}>c_{h}|t>\hat{\tau }\right)\leq \alpha .$$

Therefore, we need to determine $c_{h}$ such that the Equation (14) is satisfied. The choice $c_{h}$ can be determined through numerical simulation. More specifically, we let the global per-comparison error rate equals α, that is,

(15) $$\frac{1}{p}\sum_{j=1}^{p}P_{H_{1t}}\left({\tilde{W}}_{jt}>c_{h}|t>\hat{\tau }\right)=\alpha .$$

It is obvious that when Equation (15) is satisfied, the Equation (14) above is naturally controlled within α and holds true.

For a given initial threshold value of $c_{h}$, (such as $c_{h}=\chi_{1-\alpha }^{2}\left(1\right)$), the proportion of ${\tilde{W}}_{jt}>c_{h}$ is denoted as $\hat{p}$ when $t>\hat{\tau }$. Then, we repeat it *B* times (B=2000), the average values of $\hat{p}$ over *B* replications can be used to approximate $\frac{1}{p}\sum_{j=1}^{p}P_{H_{1t}}\left({\tilde{W}}_{jt}>c_{h}|t>\hat{\tau }\right)=\alpha$. Conversely, if Equation (15) is satisfied, we use numerical methods to obtain an approximate value, denoted as ${\hat{c}}_{h}$. Therefore, if ${\tilde{W}}_{jt}$ is not larger than ${\hat{c}}_{h}$, we can control the global per-comparison error rate at a level α, otherwise if ${\tilde{W}}_{jt}$ is larger than ${\hat{c}}_{h}$, this indicates that the *j*-th component of the data stream is abnormal.

### 2.5. Algorithm Steps

The main algorithm is summarized as the following steps.
**Algorithm 1:** The main algorithm steps of monitoring and diagnosis   OnlineMonitoringStage1    Given in-control data streams $X_{\mathrm{ic}}$;2    Calculate the sample mean ${\bar{X}}_{\mathrm{ic}}$ of the IC observations;3    Observe *p*-dimensional data stream $X_{t}$, the EWMA statistic as
calculate      ${\tilde{Y}}_{t}=\gamma \left(X_{t}-{\bar{X}}_{\mathrm{ic}}\right)+\left(1-\gamma \right)Y_{t-1},\;\;t=1,2,\ldots$;4    Construct global monitoring statistic ${\tilde{M}}_{t}=\max_{1\leq j\leq p}\frac{{\tilde{y}}_{jt}^{2}}{\frac{\gamma }{2-\gamma }{\hat{\sigma }}_{jj}}$;5    If ${\tilde{M}}_{t}>L_{\alpha }$, then raise the OC alarm, the estimated change point is      $\hat{\tau }=\inf\left\{t:t>t_{\mathrm{ic}}|{\tilde{M}}_{t+i}>L_{\alpha },\;i=0,1,\ldots ,n\right\}$.   FaultDiagnosisStage1    Collect *n* observations after the OC alarm signal occurs;2    Construct diagnostic statistic ${\tilde{W}}_{jt}=\frac{{\bar{y}}_{jt}^{2}}{\frac{\gamma }{n(2-\gamma )}{\hat{\sigma }}_{jj}}$,   $j=1,\ldots ,p,\;t=1,2,\ldots ,\hat{\tau }+n$;3    Record the observations set with ${\tilde{M}}_{t}\leq L_{\alpha }$ for $t=1,2,\ldots ,\hat{\tau }+n$ as *H*,      let $\hat{p}=\frac{1}{p}\sum_{j=1}^{p}P_{H_{0t}}\left({\tilde{W}}_{jt}>c_{h}|{\tilde{M}}_{t}>L_{\alpha }\right)$;4    Randomly sample *B* times from data set *H*, the average of
$\hat{p}$ over *B* replications      is used to approximate ${\hat{c}}_{h}$;5    Obtain ${\hat{J}}_{t}=\left\{j:{\tilde{W}}_{jt}>{\hat{c}}_{h}|{\tilde{M}}_{t}>L_{\alpha }\right\}$,
the diagnosed variables are the ones      corresponding to the indicators of ${\hat{J}}_{t}$.

### 2.6. Performance Evaluation Measures

In the online monitoring phase, in order to measure the performance of the proposed monitoring method, we choose the following three metrics: The first is the accuracy of the change point estimation, that is, when the estimated value $\hat{\tau }$ is close to the real change point τ, the change point estimation is accurate. The second is the type-I error rate of online monitoring, which is the proportion of normal observations mistakenly identified as outliers. The last one is the power value of online monitoring, that is, the proportion of abnormal observations that are accurately identified. The type-I error rate can be well controlled; meanwhile, the larger of the power value, approaching 100%, the better of the proposed monitoring method.

In the fault diagnosis stage, we define two relevant metrics to verify the effectiveness of the method: One is the false positive rate (FPR), a measure of the proportion of not-faulty variables that are incorrectly identified as faulty variables. The other is the true positive rate (TPR), defined as the percentage of the ratio of the number of variables that are correctly detected as faulty over the number of all not-faulty variables.

Without loss of generality, after an change point τ, the IC components of the stream are denoted as $J^{IC}=\left\{j:j=1,2,\ldots ,p_{0}\right\}$, and the OC components set is $J^{OC}=\left\{j:j=p_{0}+1,p_{0}+2,\ldots ,p\right\}$. At any time point *t*, the estimated OC components are defined as ${\hat{J}}_{t}=\left\{j:{\tilde{W}}_{jt}>{\hat{c}}_{h}|{\tilde{M}}_{t}>L_{\alpha }\right\}$. Thus, the TPR and FPR values at time point *t* are defined as

$$TPR_{t}=\frac{|{\hat{J}}_{t}⋂J^{OC}|}{|J^{OC}|}\cdot 100\%;\;\;\;FPR_{t}=\frac{\max\{0,|{\hat{J}}_{t}-J^{OC}|\}}{|J^{IC}|}\cdot 100\%,$$

where |A| represents the number of elements in set *A*. Correspondingly, up to time point *t*, the average TPR and FPR values can be defined as follows:

$$TPR=\frac{1}{t-\hat{\tau }}\sum_{i=\hat{\tau }+1}^{t}TPR_{i};\;\;\;\;FPR=\frac{1}{t-\hat{\tau }}\sum_{i=\hat{\tau }+1}^{t}FPR_{i}.$$

In fact, we should ensure that the higher the average TPR value, the better; meanwhile, we should also ensure that the higher the FPR value, the better the performance.

## 3. Simulation Studies

In this section, we conduct a comprehensive simulation study to evaluate the performance of our proposed monitoring and diagnosis framework, comparing it with several existing methodologies. For the experimental setup, we generate IC data streams from a multivariate normal distribution $N(\mu_{0},\Sigma_{p})$. To assess the effectiveness of our approach, we consider two distinct OC scenarios characterized by mean shifts:

(i) Mean abrupt shift: the OC stream follows $N(\kappa \mu_{1},\Sigma_{p})$, where κ is a parameter used to measure the degree of drift, and $\mu_{1}$ is a *p*-dimensional mean vector.

(ii) Mean gradual shift: the OC stream follows $N(\kappa \mu_{t},\Sigma_{p})$, where the mean vector $\mu_{t}$ evolves progressively over time according to the relationship, $\mu_{t+1}=\mu_{t}+\delta_{t}$, with $\delta_{t}$ representing a small incremental change vector over time.

The two mean shift scenarios described above may impact only a subset of dimensions within the data. In the case of an abrupt mean shift, it is assumed that the initial mean vector $\mu_{0}$ is equal to 0, while the post-shift mean vector $\mu_{1}$ is a sparse *p*-dimensional vector. Specifically, most of its components remain zero, with only a few components assuming a value of 1. In the context of a gradual mean shift, the change in the mean vector is similarly sparse. That is, the incremental change $\delta_{t}$ affects only a few components, while the majority of its elements remain zero. The duration of the mean shift in each affected dimension is denoted by *d*, and the magnitude of the change at each time point is given by k/d, reflecting a linear trend of increase or decrease. Over time, after a period of continuous gradual drift, the mean vector stabilizes, and the data stream enters a post-drift phase.

In both scenarios, the abnormal change occurs at a specific time point τ. Prior to τ, (i.e, when t<τ), the process remains IC. However, for t>τ, the mean values of $p_{s}$ components undergo a change, while the remaining $p-p_{s}$ components remain unchanged. This sparsity in the mean shift is a critical characteristic of high-dimensional data streams, where changes are often localized to a small subset of dimensions.

The covariance matrix $\Sigma_{p}$, which characterizes the correlation among *p*-dimensional random variables, plays a crucial role in the simulation study. In the experimental design, we maintain the assumption that $\Sigma_{p}$ remains invariant before and after the change point. Specifically, we investigate three distinct covariance matrix structures:

(a) Independent structure case: $\Sigma_{p}=I_{p\times p}$, where $I_{p\times p}$ represents the p×p identity matrix, indicating no correlation between variables;

(b) Long-range dependence structure case: $\Sigma_{p}={\left(\rho^{\left|k-l\right|}\right)}_{p\times p}$ for k,l=1,…,p, with the correlation coefficient ρ fixed at 0.5. This structure exhibits slowly decaying correlations between variables;

(c) Short-range correlation case: $\Sigma_{p}$ is a block-diagonal matrix, within each block matrix, $\Sigma_{b}={\left(\rho^{\left|k-l\right|}\right)}_{b\times b}$, k,l=1,…,b, where *b* is the block size and ρ=0.5, b=10.

The method presented in this study is fundamentally grounded in extreme value theory (EVT), henceforth referred to as the EVT method. To comprehensively evaluate its efficacy, we conducted comparative simulations with three alternative approaches, assessing their respective monitoring and/or diagnostic capabilities. The first comparative method employs an adaptive principal component (APC) selection mechanism utilizing hard-thresholding techniques, as proposed by Samaneh et al. [2]. The second method, developed by Ahmadi and Mohsen [5], incorporates a two-stage detection system combining a single Hotelling’s $T^{2}$ control chart with a Shewhart control chart, subsequently denoted as the AM method. The third approach, proposed by Aman et al. [27], presents a fault detection framework utilizing Slow Feature Analysis (SFA), specifically designed for time series models and SPC. In this study, we perform a thorough performance comparison with the Dynamic Slow Feature Analysis (DSFA) method, establishing a robust evaluation framework for our proposed methodology.

In the simulation framework, these methods are systematically applied to monitor continuous data streams and detect anomalies. The comparative analysis is performed through extensive computational experiments, with all performance metrics calculated from 1000 independent simulation runs to ensure statistical reliability and robustness.

Table 1 presents the simulated type-I error rates ($\tilde{\alpha }$) and power values ($\tilde{\beta }$) obtained from the proposed online monitoring procedure under various model configurations. The simulation was conducted with parameters set at p=200, $n_{ic}=150$, t=500, τ=200, α=0.05, and the duration of the mean shift d=30 in Scenario (ii), while examining three levels of abnormality degree κ=1.5, 2.0, 2.5, respectively. The results demonstrate that when t>τ and the proportion of abnormal data stream components ($p_{s}/p$) ranges from 10% to 25%, the empirical type-I error rates are effectively maintained around the nominal level of 5%, in most scenarios. Furthermore, the power values exhibit a positive correlation with the abnormality degree (κ), indicating that higher degrees of abnormality facilitate easier detection of anomalous data. Notably, in Scenario (i), the power values consistently exceed 99% across all models and proportions when κ≥2.0, demonstrating the method’s exceptional detection capability for abrupt changes. In contrast, Scenario (ii) shows relatively lower power values ranging from 86.6% to 94.0%, reflecting the increased challenge in detecting gradual changes compared with abrupt mutations. The standard deviations, presented in parentheses, indicate the stability of the monitoring results across different experimental conditions. These findings collectively validate the effectiveness of the proposed method in maintaining controlled type-I error rates while achieving high power across various abnormal data scenarios.

**Figure 2:**
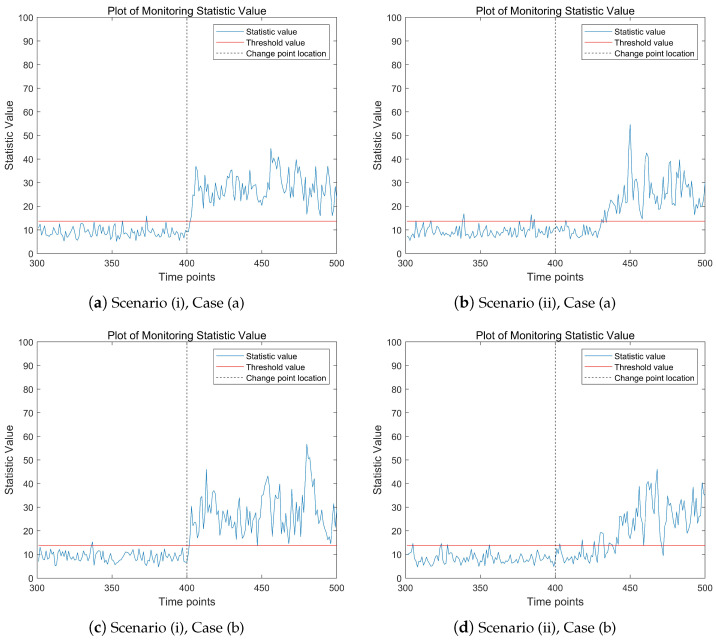

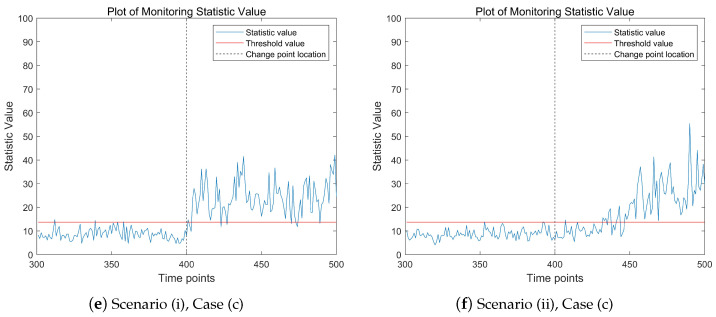
The change trend of the monitoring statistic value when p=200, $p_{s}=10$, τ=400, t=500, κ=2.5. The red dashed line represents the threshold line; the vertical gray dotted line is the change point location.

Figure 2 shows the change trend of the proposed monitoring statistics over time points in different model scenarios. The proportion of abnormal data stream is 5%. The three subfigures on the left depict the mean abrupt shift model, while the three subplots on the right represent the mean gradual shift model. In the high-dimensional data stream being monitored, the real change point time τ=400. From the results presented in Figure 2, it is evident that in all three model scenarios, the values of the monitoring statistics exhibit a significant upward trend after the change point occurs. Notably, the monitoring statistic values in the left subplots show a more pronounced upward trend post-change point compared with the right subplots. This is attributed to the abrupt mean shift in the left subplots, which leads to a more substantial increase in the monitoring statistic values. It is important to note that before the change point occurs, the values of the monitoring statistics may occasionally exceed the threshold at certain time points. This phenomenon is likely caused by outliers in the data. Overall, Figure 2 demonstrates that the proposed monitoring statistic effectively captures the changing trends in high-dimensional data streams, providing a reliable method for detecting shifts in the data.

Table 2 presents a comprehensive comparison of three change point detection methods (EVT, A-M, and APC) under different scenarios and parameter settings. The evaluation is based on three key metrics: Bias (the absolute deviation between estimated and true change points), Sd (the standard deviation of estimators), and $P_{j}$ (the probability that the absolute difference between true and estimated change points is within a specified threshold *j*). The results demonstrate a clear trend across all methods: as the signal strength parameter κ increases from 1.0 to 2.0, the estimation accuracy improves significantly. This improvement is evidenced by the decreasing values of Bias and Sd, along with the increasing values of $P_{j}$. Specifically, when κ reaches 2.0, all methods achieve their best performance, with $P_{j}$ values approaching or exceeding 90% in most cases.

In Scenario (i) with τ=200, the EVT method shows superior performance, particularly at higher κ values. When κ=2.0, EVT achieves a remarkably low Bias of 1.1 and Sd of 1.1, with $P_{3}$ reaching 99.5%. The APC method also demonstrates competitive performance, showing consistent improvement across different κ values. The A-M method, while showing improvement with increasing κ, generally underperforms compared with the other two methods in this scenario. For Scenario (i) with τ=400, similar patterns emerge, though the absolute values of the metrics are slightly different. The EVT method maintains its leading position, achieving a Bias of 1.2 and Sd of 0.8 at κ=2.0, with $P_{3}$ remaining at 99.5%. The APC method shows particularly strong improvement in this scenario, with $P_{3}$ increasing from 57.1% at κ=1.0 to 92.7% at κ=2.0. In Scenario (ii), which represents a more challenging detection environment, all methods show higher Bias and Sd values compared with Scenario (i). However, the relative performance ranking remains consistent, with EVT maintaining its advantage, followed by APC and then A-M. Notably, even in this more difficult scenario, the EVT method achieves $P_{15}$ values above 90% when κ reaches 2.0.

These results collectively demonstrate that while all methods benefit from stronger signals (higher κ values), the EVT method consistently outperforms the others across different scenarios and parameter settings. The findings suggest that the choice of detection method should consider both the expected signal strength and the required precision of change point estimation.

The size of the smoothing parameter γ plays a crucial role in determining the performance of the EWMA-based monitoring procedure, particularly affecting the type-I error rate, detection power, and change point estimation accuracy. Table 3 presents a comprehensive simulation study evaluating these performance metrics under different γ values (0.2, 0.4, and 0.6) across various abnormality indicators (κ = 1.0, 1.5, 2.0) and sparsity levels ($p_{s}/p$ = 0.05, 0.10, 0.15) of the mean abrupt shift model. The simulation results reveal several important patterns. First, the type-I error rate remains stable around the nominal level of 5% across all γ values, demonstrating the robustness of the proposed method in maintaining false alarm control. However, the power $\tilde{\beta }$ shows a strong dependence on γ, with higher values of γ leading to substantially reduced detection rates. For instance, when κ = 1.0 and $p_{s}/p=0.05$, the detection power decreases from 80.4% at γ = 0.2 to only 17.8% at γ = 0.6.

The change point estimation accuracy, measured by $\hat{\tau }$, also exhibits sensitivity to γ selection. Smaller γ values consistently provide more precise change point detection, particularly for smaller mean shifts (κ = 1.0). For example, at $p_{s}/p=0.05$ and κ = 1.0, the estimation error increases from 9.5 (209.5–200) at γ = 0.2 to 90.1 at γ = 0.6. This pattern is particularly pronounced for smaller κ values, confirming the EWMA statistic’s sensitivity to small mean drifts. Interestingly, as κ increases, the influence of γ on both detection power and change point estimation diminishes. At κ = 2.0, even with γ = 0.6, the method achieves detection power above 85% across all sparsity levels, and the change point estimation error remains within 5.5 time units. This suggests that while γ selection is critical for detecting small changes, its impact becomes less significant when dealing with larger mean shifts. The results also indicate that higher sparsity levels ($p_{s}/p$) generally improve detection performance, particularly for smaller γ values. This pattern is most evident in the κ = 1.0 scenario, where increasing $p_{s}/p$ from 0.05 to 0.15 improves detection power from 80.4% to 95.9% at γ = 0.2.

These findings collectively suggest that while smaller γ values (around 0.2) are preferable for detecting small changes and achieving accurate change point estimation, larger γ values may be more suitable when robustness to noise is prioritized over sensitivity to small shifts. The choice of γ should therefore be guided by the specific monitoring objectives and the expected magnitude of process changes.

**Figure 3:**
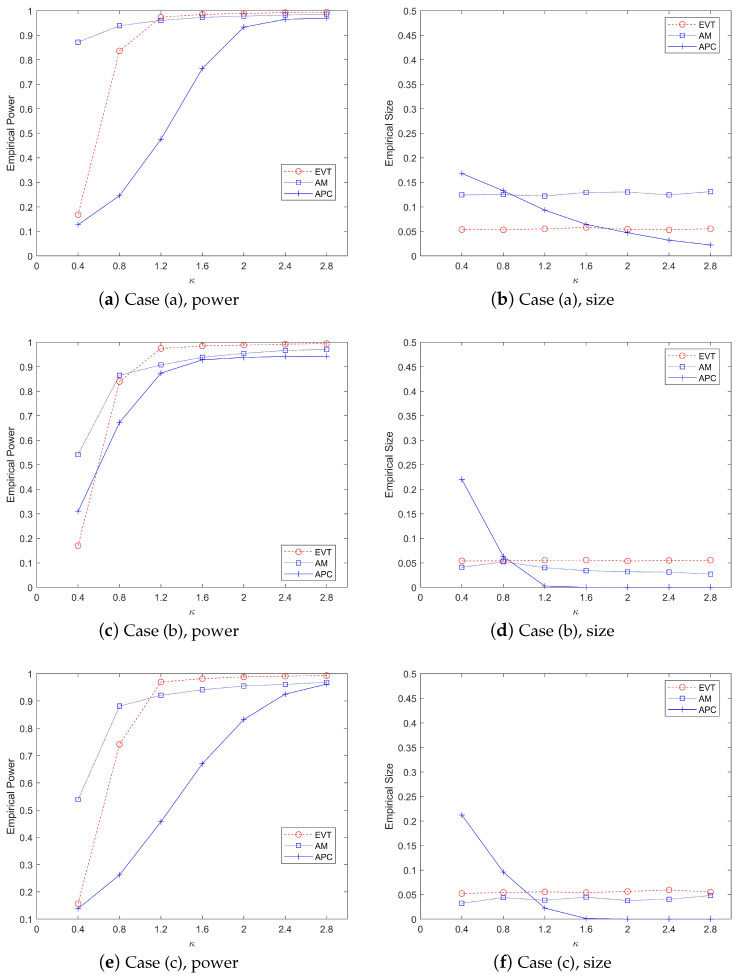
The change trend of power and size values (%) by various procedures when p=200, $p_{s}=20$, τ=200, t=300, α=0.05 with the increase in the parameter κ.

Figure 3 illustrates a comparative analysis of the performance metrics, including power values and type-I error rates, across three monitoring methods under varying κ parameter values, evaluated against three distinct covariance matrix structures in the mean abrupt shift scenario. The left panel of the figure demonstrates the power values obtained by each method. When κ = 0.4 or κ = 0.8, the AM method exhibits a slight advantage over our proposed EVT method. However, as κ increases to 1.2 or beyond, the EVT method significantly outperforms both the AM and APC methods in terms of power values. The right panel of Figure 3 focuses on the type-1 error rates. It is evident that the EVT method effectively controls the type-1 error rate around the nominal level of 5%. In contrast, the AM method fails to maintain control over the type-I error rate. The APC method also shows suboptimal performance in controlling type-I errors. Overall, Figure 3 highlights the robustness and superiority of the EVT method, especially for larger values of κ, both in terms of power and type-I error control. The results underscore the limitations of the AM and APC methods in maintaining statistical control under varying conditions.

Table 4 lists the fault diagnosis results of high-dimensional data streams by using different diagnostic methods. We have calculated the values of two main indicators here: one is the correct recognition rate, and the other is the incorrect recognition rate. At the same time, we also considered the value of change point estimation during the online monitoring phase. The results show that as *k* increases, the change point estimation becomes closer to the true value. Regardless of the proportion of $p_{s}$ being equal to 5%, 10%, or 15%, the EVT method is significantly better than the other two methods. Furthermore, from the diagnostic results, the TPR values of the EVT method are close to 100%. It should be noted that the AM method and APC method also have good diagnostic results. As pointed out by Vilenchik et al. (2019) [35], PCA-based approaches, such as APC, face a problem of difficult interpretation, especially for high-dimensional data. Considering this, as well as the results of point estimation, our method is, overall, superior to the other two methods.

Recently, Aman et al. [27] proposed a novel methodology for fault detection based on Slow Feature Analysis (SFA), specifically tailored for time series models and SPC. Their comprehensive analysis demonstrates that Kernel SFA (KSFA) and Dynamic SFA (DSFA) significantly outperform traditional methods by offering enhanced sensitivity and fault detection capabilities.

**Figure 4:**
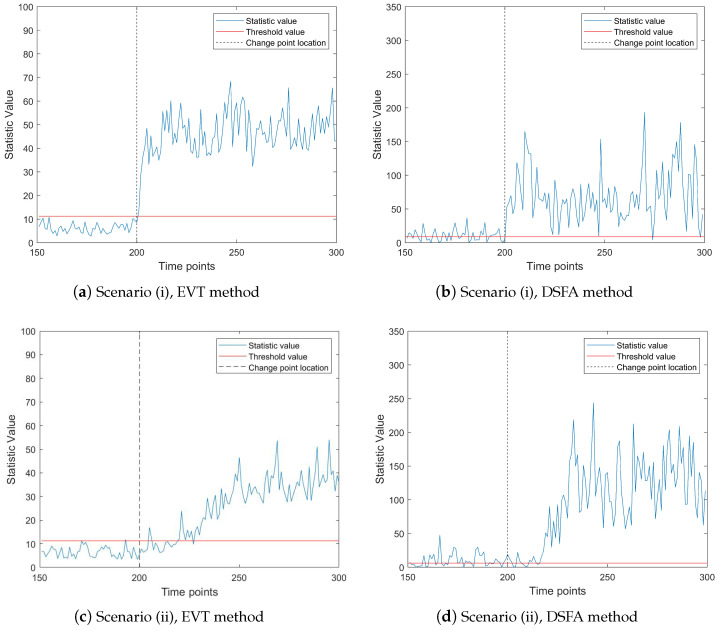
The change trend of the monitoring statistic value when p=50, $p_{s}=30$, τ=200, $n_{ic}=150$, t=300, κ=2.5. The red dashed line represents the threshold line; the vertical gray dotted line is the change point location.

**Figure 5:**
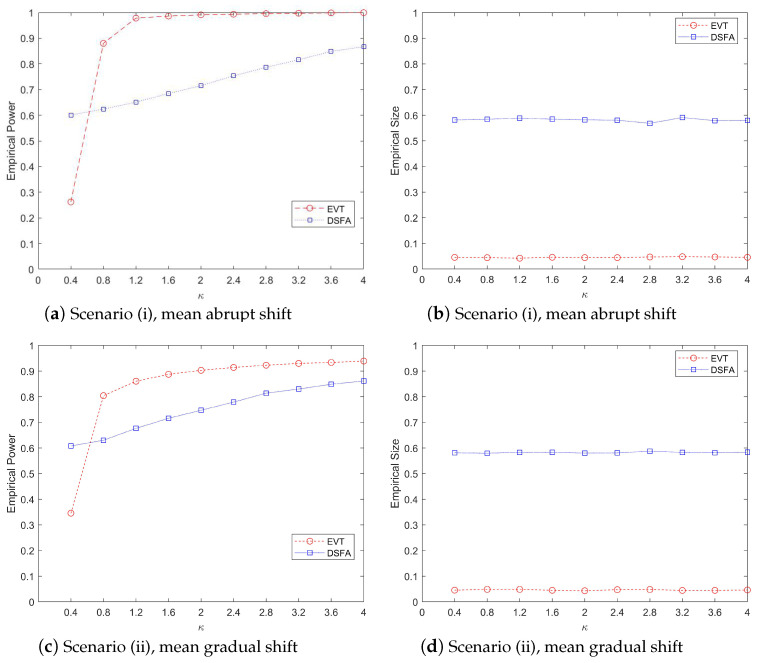
The change trend of power and size values (%) by various procedures when p=200, $p_{s}=20$, τ=200, t=300, α=0.05 with the increase in the parameter κ.

To further evaluate the effectiveness of fault detection methods, we compare the proposed EVT online monitoring method with the DSFA method. The results of this comparison are presented in Figure 4 and Figure 5. It is important to note that, as observed in prior analyses, the DSFA method struggles to effectively monitor high-dimensional and ultra-high-dimensional sparsely changing time series data streams, primarily due to the challenges posed by the curse of dimensionality. To facilitate a more meaningful comparison, we select a data stream with a slightly lower dimensionality. Specifically, we set the parameters as follows: p=50, t=300, $n_{ic}=150$, τ=200, with 30 components exhibiting abnormal changes. We examine two scenarios of mean change: (i) mean abrupt shift and (ii) mean gradual shift. In the gradual shift scenario, the duration of the change is set to d=30 time units.

Figure 4 illustrates the performance of both the EVT and DSFA methods under these two scenarios. The left subfigure depicts the mean abrupt shift case, while the right subfigure represents the mean gradual shift case. In both subfigures, the gray lines denote IC data, and the red lines indicate OC data. From Figure 4, it is evident that both methods exhibit significant changes in monitoring statistics after the change point, indicating their ability to detect faults. In the first scenario (mean abrupt shift), the EVT method promptly reflects the abnormal changes in the data stream, with the monitoring statistics clearly exceeding the threshold at the change point. Moreover, in the absence of changes, most of the monitoring statistics remain below the threshold, demonstrating the method’s robustness. In the second scenario (mean gradual shift), both methods exhibit a delay in detection due to the gradual nature of the change. However, the DSFA method incorrectly identifies a substantial amount of normal data as abnormal before the change point occurs, highlighting its limitations in handling gradual shifts. In contrast, the EVT method demonstrates superior performance by more accurately distinguishing between normal and abnormal data, even in the presence of gradual changes.

Overall, the results in Figure 4 underscore the advantages of the EVT method over the DSFA method, particularly in terms of timely and accurate fault detection in both abrupt and gradual change scenarios. This comparison reinforces the effectiveness of the EVT approach for online monitoring of time series data streams.

Figure 5 illustrates the change trends in monitoring power and type-I error rates for the EVT and DSFA methods as the degree of abnormality κ increases from 0.4 to 4.0. The figure examines two types of mean changes: abrupt and gradual shifts. The top two subplots (a) and (b) depict the performance of the two methods in terms of monitoring power and type I error under abrupt mean changes, while the bottom two subplots (c) and (d) show the corresponding trends under gradual mean changes.

From Figure 5, it is evident that the monitoring power of both methods increases with the rise in κ, as shown in the left subplots. However, the EVT method consistently outperforms the DSFA method, demonstrating superior monitoring efficiency. Specifically, the power values monitored by the EVT method are significantly higher than those of the DSFA method, regardless of whether the mean change is abrupt or gradual. This indicates that the EVT method is more effective in detecting anomalies as κ increases. The right subplots focus on the type-I error rates. The EVT method effectively controls the error rate around the 5% threshold, maintaining reliability in false positive detection. In contrast, the DSFA method struggles to control the type-I error, with many monitoring statistics exceeding the threshold before the change point occurs. This aligns with the results observed in Figure 4, further highlighting the robustness of the EVT method in managing error rates. In summary, Figure 5 demonstrates that while both methods improve in monitoring power with increasing κ, the EVT method is significantly more effective and reliable, particularly in controlling type-I errors, making it a preferable choice for detecting mean changes in data streams.

## 4. Real Data Analysis

To validate the effectiveness of the proposed methodology, we apply our monitoring and diagnostic framework to a real-world industrial dataset involving archaeological glass vessel analysis. This dataset simulates a typical high-dimensional process monitoring scenario in material manufacturing industries. The data originates from Electron Probe X-ray Microanalysis (EPXMA) measurements of sixteenth- to seventeenth-century glass artifacts [36]. Previous studies have extensively analyzed this dataset for anomaly detection, including Hubert et al. (2005) [37], who applied RobPCA and identified row-wise outliers, and Hubert et al. (2019) [38], who employed MacroPCA to flag anomalous samples.

In industrial practice, EPXMA serves as a critical tool for quality control in glass production, semiconductor manufacturing, and metallurgy, where real-time monitoring of material composition ensures product consistency and enables early anomaly detection. For instance, modern glass manufacturing requires precise detection of elemental composition deviations caused by raw material impurities or furnace temperature fluctuations, which could otherwise lead to defective batches. Our case study treats the glass artifact data as a high-dimensional production process stream, containing 180 sequential time points (analogous to production cycles) and 750 spectral intensity variables. These EPXMA intensities reflect elemental composition characteristics comparable to real-time spectroscopic monitoring in industrial production lines. Each time point corresponds to a manufactured batch, with variables representing critical quality indicators such as SiO_2_, CaO, and trace element concentrations.

During preliminary analysis, we excluded 13 variables with missing values or measurement artifacts (e.g., sensor dropout events), retaining 737 process variables. This preprocessing aligns with industrial protocols for dynamically excluding faulty sensors from monitoring systems. Shapiro-Wilk normality tests reveal non-normal distributions for most components (*p*-values < 0.01). Following previous studies, we resampled the first 30 IC observations 100 times to generate a 3000-sample IC reference set. All IC observations served as historical data, with 180 subsequent observations used for testing. To approximate normality assumptions, we applied an inverse normal transformation: $\Phi^{-1}\left({\hat{F}}_{j}\left(X_{jt}\right)\right)$ for j=1,…,737, t=1,…,180, where ${\hat{F}}_{j}\left(X_{jt}\right)$ denotes the empirical cumulative distribution function for the *j*-th IC component. Notably, this marginal transformation does not enforce joint normality.

**Figure 6:**
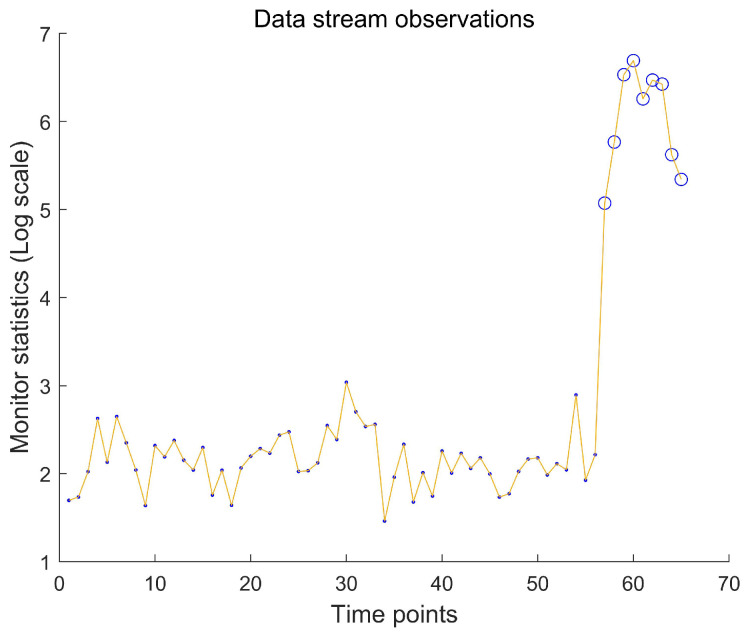
Online monitoring results for the glass production process stream. Solid points: IC batches; Hollow circles: OC batches triggering alarms. The OC state from t=57 onward emulates a production line deviation requiring intervention.

We implement our online monitoring framework with a control limit α=0.05 and weight parameter γ=0.4. As shown in Figure 6, the system detects an OC state at t=57, indicating a systemic process deviation. This finding corroborates Hubert et al. (2019) [38], who identified similar anomalies using offline Robust PCA methods. In industrial practice, such detection would trigger immediate production checks for potential causes like raw material contamination or equipment malfunctions.

In the second stage, to identify the root causes of data stream anomalies, we perform a detailed analysis of observations within a defined window (ω = 5), covering five consecutive time points (58–62) after the change point. By using the fault diagnosis Algorithm 1 proposed in this study, we demonstrate significant intensity shifts in 36 variables, suggesting potential anomalies. While the specific chemical elements corresponding to the spectral data remain unidentified, we postulate that these shifts may indicate variations in certain chemical components, particularly those related to potassium (K) and lead (Pb) oxides. In an industrial setting, this precise diagnostic capability facilitates targeted quality adjustments for specific deviations, such as changes in K/Pb ratios due to contaminated raw materials. This diagnostic method marks a substantial improvement over conventional approaches that simply identify ‘abnormal batches’ without offering actionable insights for operational adjustments.

## 5. Conclusions and Discussion

This study develops a statistically rigorous framework for online monitoring and fault diagnosis in high-dimensional data streams with sparse anomalies. The proposed two-stage methodology integrates an EWMA-based monitoring scheme with the diagnostic Algorithm 1, demonstrating quantifiable improvements over conventional approaches. Numerical simulations validate that the monitoring stage achieves rapid anomaly detection (typically within 1 or 2 sampling intervals post-change) while maintaining type-I error rates at approximately 5%, aligning with conventional control limits. The diagnostic stage exhibits exceptional precision, attaining 99.1% identification accuracy for 200-dimensional anomaly streams and reducing false discovery rates to below 3–28.0% improvement over conventional approaches. These quantifiable advancements address critical limitations in existing methods, particularly in balancing sensitivity to sparse changes with stringent error control. This method demonstrates effectiveness in monitoring high-dimensional data streams characterized by abnormal mean changes.

However, its capability to detect anomalies is limited in scenarios involving covariance changes, indicating certain constraints in capturing diverse types of abnormal patterns. Consequently, the proposed approach imposes specific requirements on data quality, particularly regarding data distribution, to satisfy its theoretical assumptions. Moreover, it should be noted that several algorithmic parameters require careful calibration based on actual data characteristics in practical applications. Specifically, the transformation parameter γ and the diagnostic window width ω need appropriate adjustment according to the data quality. Future research should focus on extending the framework to address these constraints. Adaptive covariance estimation techniques and robust statistical formulations could enhance robustness to non-Gaussian noise and covariance shifts. Computational optimizations during initialization may improve scalability for ultra-high-dimensional systems. Furthermore, investigating adaptive parameter selection strategies and relaxing independence assumptions could broaden applicability to correlated anomaly patterns. Despite these limitations, the proposed method establishes a principled foundation for high-dimensional stream analysis, particularly in applications demanding sparse fault detection with controlled FDR.

## Figures and Tables

**Table 1:** Average percentage of the empirical type-I error values $\tilde{\alpha }$ (%) and the power values $\tilde{\beta }$ (%) by the proposed monitoring procedure under different model settings when p=200, $n_{ic}=150$, t=500, τ=200, α=0.05, and κ=1.5, 2.0, 2.5, respectively.

Scenario	Model		κ = 1.5		κ = 2.0		κ = 2.5	
		$p_{s}/p$	$\tilde{\alpha }$	$\tilde{\beta }$	$\tilde{\alpha }$	$\tilde{\beta }$	$\tilde{\alpha }$	$\tilde{\beta }$
	(a)	0.05	5.3(3.4)	99.2(0.5)	5.4(3.4)	99.7(0.2)	5.5(3.5)	99.9(0.2)
		0.10	5.2(3.4)	99.7(0.2)	5.4(3.4)	99.8(0.7)	5.5(3.4)	99.9(0.1)
		0.20	5.6(3.6)	99.8(0.2)	5.6(3.1)	99.9(0.1)	5.7(3.6)	100.0(0.1)
(i)	(b)	0.05	5.5(3.2)	96.6(1.4)	5.4(3.6)	99.7(0.2)	5.2(3.2)	99.9(0.2)
		0.10	5.4(3.4)	99.5(0.3)	5.3(3.7)	99.8(0.2)	4.9(3.4)	99.9(0.1)
		0.20	5.4(3.6)	99.7(0.2)	5.3(3.5)	99.8(0.2)	5.3(3.3)	100.0(0.1)
	(c)	0.05	4.9(3.1)	96.7(1.3)	5.3(3.3)	99.7(0.2)	5.3(3.2)	99.9(0.2)
		0.10	5.1(3.5)	99.6(0.3)	5.3(3.6)	99.8(0.2)	5.4(3.5)	99.9(0.1)
		0.20	5.1(3.2)	99.7(0.2)	5.4(3.5)	99.9(0.2)	5.4(3.4)	100.0(0.1)
	(a)	0.05	5.6(4.3)	87.7(1.5)	5.5(4.2)	90.4(1.2)	5.5(3.9)	92.9(1.0)
		0.10	5.4(3.9)	89.7(1.2)	5.5(4.1)	91.6(1.0)	5.3(3.8)	93.9(0.9)
		0.20	5.4(4.0)	90.9(1.1)	5.7(4.1)	92.9(1.0)	5.2(3.9)	94.0(0.9)
(ii)	(b)	0.05	5.3(3.9)	86.6(1.8)	5.2(3.9)	89.6(1.4)	5.3(3.6)	92.5(1.2)
		0.10	5.5(3.9)	88.7(1.3)	5.3(4.0)	91.1(1.2)	5.1(4.2)	92.9(1.1)
		0.20	5.4(3.9)	90.3(1.2)	5.6(4.0)	92.4(1.1)	5.3(4.1)	93.6(0.9)
	(c)	0.05	5.3(4.1)	86.7(1.7)	5.3(3.8)	89.7(1.6)	5.2(3.6)	91.6(1.2)
		0.10	5.4(4.1)	88.7(1.3)	5.3(4.0)	91.8(1.3)	5.2(3.9)	92.7(1.1)
		0.20	5.2(3.8)	90.3(1.2)	5.5(4.2)	92.3(1.1)	5.4(4.0)	93.6(0.9)

**Table 2:** Comparison of the three methods via change point estimation with p=200, $p_{s}=40$, t=500, α=0.05, the real change point τ=200 and 400, respectively. Bias: the absolute deviation of estimators; Sd: the standard deviations of estimators; $P_{j}=100\mathrm{Pr}(|\tau -\hat{\tau }\left|\leq j\right)$%, j=3 in Scenario (i), and j=15 in Scenario (ii).

			τ = 200			τ = 400		
Scenario	Method	κ	Bias	Sd	$P_{j}$	Bias	Sd	$P_{j}$
	EVT	1.0	6.3	3.8	24.0	7.1	5.0	23.6
		1.5	2.1	1.2	89.0	2.2	1.2	88.5
		2.0	1.1	1.1	99.5	1.2	0.8	99.5
	A-M	1.0	9.4	4.5	13.5	6.8	5.4	35.2
(i)		1.5	6.1	2.8	17.5	4.5	3.6	37.3
		2.0	4.1	2.1	34.0	3.2	2.5	44.0
	APC	1.0	4.6	3.1	44.9	3.6	2.7	57.1
		1.5	3.6	1.7	55.0	2.5	1.3	79.0
		2.0	3.1	1.2	70.2	2.1	0.9	92.7
	EVT	1.0	22.1	3.5	3.9	21.7	12.1	3.5
		1.5	15.8	2.7	43.7	15.3	10.4	42.1
		2.0	12.3	3.08	91.0	12.4	8.1	90.3
	A-M	1.0	18.8	7.1	17.2	14.2	10.1	36.2
(ii)		1.5	14.9	6.1	34.2	12.3	7.8	47.1
		2.0	13.3	4.9	64.9	9.9	7.1	74.1
	APC	1.0	16.0	3.3	49.5	14.7	4.6	57.8
		1.5	15.2	2.9	59.0	13.8	3.7	65.5
		2.0	14.7	2.3	76.1	13.2	2.5	80.2

**Table 3:** Average percentage of type-I error values ($\tilde{\alpha }$%), power values ($\tilde{\beta }$%), and change point estimators ($\hat{\tau }$) by the proposed online monitoring procedure under different choices of γ when p=200, t=300, τ=200, and α=0.05.

		κ = 1.0			κ = 1.5			κ = 2.0		
$p_{s}/p$	γ	$\tilde{\alpha }$	$\tilde{\beta }$	$\hat{\tau }$	$\tilde{\alpha }$	$\tilde{\beta }$	$\hat{\tau }$	$\tilde{\alpha }$	$\tilde{\beta }$	$\hat{\tau }$
	0.2	4.8	80.4	209.5	4.9	97.1	202.8	4.9	98.4	201.4
0.05	0.4	4.9	34.7	265.1	5.0	83.4	207.3	4.9	98.3	201.5
	0.6	5.0	17.8	290.1	5.0	50.3	248.1	4.9	85.9	205.2
	0.2	5.0	92.9	206.0	5.0	97.9	201.6	4.9	98.8	200.8
0.10	0.4	5.0	53.9	232.9	4.9	95.7	202.5	5.0	99.2	200.8
	0.6	4.9	28.1	292.5	5.0	73.4	213.1	4.9	97.4	201.5
	0.2	5.0	95.9	204.1	5.0	98.3	201.5	4.9	99.0	200.4
0.15	0.4	4.9	67.1	215.6	5.0	98.2	201.6	4.9	99.2	200.6
	0.6	5.0	37.3	281.7	5.0	84.7	205.5	4.9	99.2	200.5

**Table 4:** Diagnosis accuracy of the proposed procedure, under different choices of κ and $p_{s}$, when p=200, t=300, τ=200, and α=0.05.

		EVT			A-M			APC		
$p_{s}$	κ	$\hat{\tau }$	TPR	FPR	$\hat{\tau }$	TPR	FPR	$\hat{\tau }$	TPR	FPR
	1.0	212.3	99.1	0.8	213.6	100.0	0.9	210.5	71.1	4.4
5%	1.5	203.3	100.0	0.8	208.6	100.0	0.9	208.1	82.1	5.4
	2.5	201.6	100.0	0.8	206.7	100.0	0.9	206.9	85.8	7.4
	1.0	206.5	99.3	1.3	209.5	100.0	0.9	209.9	84.8	10.3
10%	1.5	202.1	100.0	1.4	206.1	100.0	1.0	206.7	92.8	12.9
	2.5	201.2	100.0	1.3	204.6	100.0	0.8	206.2	94.4	15.0
	1.0	204.5	99.5	1.6	209.8	100.0	0.6	208.1	91.8	12.6
15%	1.5	201.4	99.7	1.7	205.0	100.0	0.9	206.2	95.7	14.2
	2.5	200.9	100.0	1.7	204.4	100.0	0.4	205.5	96.1	13.4

## Data Availability

The raw data supporting the conclusions of this paper will be made available by the authors upon request.

## Abbreviations

The following abbreviations are used in this manuscript:

SPC	statistical process control
EWMA	exponentially weighted moving average
CUSUM	cumulative sum
IC	in-control
OC	out-of-control
ARL	average run length
FDR	false discovery rate
PCER	per-comparison error rate
FPR	false positive rate
TPR	true positive rate
EVT	extreme value theory
DSFA	dynamic slow feature analysis
PCA	principal component analysis
APC	adaptive principal component

## References

[1] Zou C., Wang Z., Zi X., Jiang W. (2015). An efficient online monitoring method for high-dimensional data streams. Technometrics.

[2] Ebrahimi S., Ranjan C., Paynabar K. (2020). Monitoring and root-cause diagnostics of high-dimensional data streams. J. Qual. Technol..

[3] Li J. (2019). A two-stage online monitoring procedure for high-dimensional data streams. J. Qual. Technol..

[4] Li W., Xiang D., Tsung F., Pu X. (2020). A diagnostic procedure for high-dimensional data streams via missed discovery rate control. Technometrics.

[5] Ahmadi-Javid A., Ebadi M. (2020). A two-step method for monitoring normally distributed multi-stream processes in high dimensions. Qual. Eng..

[6] Liu K., Mei Y., Shi J. (2015). An adaptive sampling strategy for online high-dimensional process monitoring. Technometrics.

[7] Huang T., Kandasamy N., Sethu H., Stamm M. (2019). An efficient strategy for online performance monitoring of datacenters via adaptive sampling. IEEE Trans. Cloud Comput..

[8] Xiang D., Li W., Tsung F., Pu X., Kang Y. (2021). Fault classification for high-dimensional data streams: A directional diagnostic framework based on multiple hypothesis testing. Nav. Res. Logist. (NRL).

[9] Li J. (2020). Efficient global monitoring statistics for high-dimensional data. Qual. Reliab. Eng. Int..

[10] Xian X., Zhang C., Bonk S., Liu K. (2021). Online monitoring of big data streams: A rank-based sampling algorithm by data augmentation. J. Qual. Technol..

[11] Colosimo B., Jones-Farmer L., Megahed F., Paynabar K., Ranjan C., Woodall W. (2024). Statistical process monitoring from industry 2.0 to industry 4.0: Insights into research and practice. Technometrics.

[12] Xiang D., Qiu P., Wang D., Li W. (2022). Reliable post-signal fault diagnosis for correlated high-dimensional data streams. Technometrics.

[13] Mutambik I. (2024). An entropy-based clustering algorithm for real-time high-dimensional IoT data streams. Sensors.

[14] Wan Y., Lin S., Jin C., Gao Y., Yang Y. (2024). Improved entropy-based condition monitoring for pressure pipeline through acoustic denoising. Entropy.

[15] Hotait H., Chiementin X., Rasolofondraibe L. (2021). Intelligent online monitoring of rolling bearing: Diagnosis and prognosis. Entropy.

[16] Wu H., Yuan R., Lv Y., Stein D., Zhu W. (2024). Multi-weighted symbolic sequence entropy: A novel approach to fault diagnosis and degradation monitoring of rotary machinery. Meas. Sci. Technol..

[17] Wang Z., Sun Y. (2023). Role of entropy in fault diagnosis of mechanical equipment: A Review. Eng. Res. Express.

[18] Hu X., Zhao Y., Yeganeh A., Shongwe S. (2024). Two memory-based monitoring schemes for the ratio of two normal variables in short production runs. Comput. Ind. Eng..

[19] Liu Y., Ren H., Li Z. (2024). A unified diagnostic framework via Symmetrized Data Aggregation. IISE Trans..

[20] Yan X., Sarkar M., Lartey B., Gebru B., Homaifar A., Karimoddini A., Tunstel E. (2023). An Online Learning Framework for Sensor Fault Diagnosis Analysis in Autonomous Cars. IEEE Trans. Intell. Transp. Syst..

[21] Chen D., Zhang Z., Zhou F., Wang C. (2024). A real-time fault diagnosis method for multi-source heterogeneous information fusion based on two-Level transfer learning. Entropy.

[22] Li H., Zheng Z., Liu K. (2025). Online Monitoring of High-dimensional Data Streams with Deep Q-network. IEEE Trans. Autom. Sci. Eng..

[23] Li D., Bai M., Xian X. (2024). Data-Driven Pathwise Sampling Approaches for Online Anomaly Detection. Technometrics.

[24] Cheruku S., Balaji S., Adolfo D., Krishnamurthy V. (2025). Data-Driven Digital Twins for Real-Time Machine Monitoring: A Case Study on a Rotating Machine. J. Comput. Inf. Sci. Eng..

[25] MacGregor J., Cinar A. (2012). Monitoring, fault diagnosis, fault-tolerant control and optimization: Data driven methods. Comput. Chem. Eng..

[26] Zhao P., Qinghe Z., Ding Z., Zhang Y., Wang H., Yang Y. (2021). A High-Dimensional and Small-Sample Submersible Fault Detection Method Based on Feature Selection and Data Augmentation. Sensors.

[27] Aman A., Chen Y., Yiqi L. (2024). Assessment of Slow Feature Analysis and Its Variants for Fault Diagnosis in Process Industries. Technologies.

[28] Yoav B., Daniel Y. (2001). A multivariate exponentially weighted moving average control chart. Ann. Stat..

[29] Lowry C.A., Woodall W.H., Champ C.W., Rigdon S.E. (1992). A multivariate exponentially weighted moving average control chart. Technometrics.

[30] Feng L., Ren H., Zou C. (2020). A setwise EWMA scheme for monitoring high-dimensional datastreams. Random Matrices Theory Appl..

[31] Cai T.T., Liu W., Xia Y. (2014). Two-sample test of high dimensional means under dependence. J. R. Stat. Soc..

[32] Yoav B., Yosef H. (1995). Controlling the false discovery rate: A practical and powerful approach to multiple testing. J. R. Stat. Soc. Ser. B (Methodol.).

[33] Benjamini Y., Yekutieli D. (2001). The control of the false discovery rate in multiple testing under dependency. Ann. Stat..

[34] Du L., Guo X., Sun W., Zou C. (2021). False discovery rate control under general dependence by symmetrized data aggregation. J. Am. Stat. Assoc..

[35] Vilenchik D., Yichye B., Abutbul M. (2019). To Interpret or Not to Interpret PCA? This Is Our Question. Proc. Int. AAAI Conf. Web Soc. Media.

[36] Lemberge P., De Raedt I., Janssens K.H., Wei F., Van Espen P.J. (2000). Quantitative analysis of 16–17th century archaeological glass vessels using PLS regression of EPXMA and *μ*-XRF data. J. Chemom. J. Chemom. Soc..

[37] Hubert M., Rousseeuw P., Branden K. (2005). ROBPCA: A new approach to robust principal component analysis. Technometrics.

[38] Hubert M., Rousseeuw P., Van den Bossche W. (2019). MacroPCA: An all-in-One PCA method allowing for missing values as well as cellwise and rowwise outliers. Technometrics.

